# Efficacy of Gut Microbiome-Targeted Interventions on Mental Health Symptoms in Women Across Key Hormonal Life Stages: A Systematic Review and Meta-Analysis of Randomized Controlled Trials

**DOI:** 10.3390/healthcare13222851

**Published:** 2025-11-10

**Authors:** Naika Dubois, Coralie Vincent, Isabelle Giroux

**Affiliations:** 1 Interdisciplinary School of Health Sciences, Faculty of Health Sciences, University of Ottawa, Ottawa, ON K1N 6N5, Canada; 2 School of Nutrition Sciences, Faculty of Health Sciences, University of Ottawa, Ottawa, ON K1N 6N5, Canada; 3 Institut du Savoir Montfort, Ottawa, ON K1K 0M9, Canada

**Keywords:** microbiome, microbiota, probiotics, prebiotics, mental health, depression, anxiety, premenstrual, perinatal, menopause

## Abstract

**Background**: Women are disproportionately affected by depression and generalized anxiety disorder compared to men throughout their lives. Hormonal changes during the menstrual cycle, pregnancy, postpartum, and menopause are often associated with mood disturbances. Evidence suggests that modulating the gut microbiome through gut-targeted interventions may offer a novel therapeutic approach for various mental health conditions. **Objective**: This systematic review and meta-analysis aimed to synthesize evidence from randomized controlled trials (RCTs) on the efficacy of gut microbiome-targeted interventions in improving mental health symptoms in women during key hormonal transitions. **Methods**: A systematic search was conducted from inception to August 2025 across Embase, MEDLINE (PubMed), Web of Science, PsycINFO, CINAHL, Scopus, FSTA, CENTRAL, the WHO International Clinical Trials Registry Platform, and ClinicalTrials.gov. Two reviewers independently screened, extracted data, and assessed study quality. Methodological quality was evaluated using Cochrane’s risk-of-bias tool (RoB 2.0). Statistical analyses were performed with Comprehensive Meta-Analysis software (version 4). **Results**: Eleven RCTs were included, of which eight were used in the meta-analyses. Gut microbiome-targeted interventions significantly reduced depressive symptoms (Standardized Mean Difference (SMD) = −0.848; 95% Confidence Interval (CI): −1.470 to −0.226; *p* = 0.008) and anxiety symptoms (SMD = −0.997; 95% CI: −1.684 to −0.311; *p* = 0.004) versus controls. Heterogeneity was high (depression: Cochran’s Q = 87.1, I^2^ = 92%, τ^2^ = 0.729; anxiety: Q = 35.3, I^2^ = 89%, τ^2^ = 0.535), but sensitivity analyses confirmed robustness. Meta-regressions indicated that treatment duration was not a significant moderator (depression: *p* = 0.12; anxiety: *p* = 0.28). **Conclusions**: Gut-targeted interventions significantly reduced symptoms of both depression and anxiety, highlighting their potential as complementary therapeutic strategies for managing mood disorders in women across hormonal life stages. However, high heterogeneity limits the ability to determine optimal standardized clinical recommendations, highlighting the need for further research to guide clinical applications and inform individualized approaches to treatment.

## 1. Introduction

Depression constitutes a major global health burden worldwide. Research indicates that women are disproportionately affected, with approximately twice the likelihood of experiencing depression and generalized anxiety disorder compared to men throughout their lives [1,2,3,4,5,6]. The period of women’s reproductive years, from early adolescence to their mid-50s, is recognized as a time of substantially increased vulnerability for the onset of depressive disorders [5,7,8,9,10]. Hormonal changes during the menstrual cycle, pregnancy, postpartum, and menopause are commonly associated with the appearance or worsening of mood disorders [5,8,9,11,12,13,14].

Growing evidence indicates that hormonal fluctuations throughout women’s life stages can influence gut microbiota composition, which in turn may affect mental health outcomes [15,16,17,18,19,20,21]. These outcomes include premenstrual syndrome (PMS), characterized by mood swings, tension, and irritability; perinatal mood disorders, often presenting with sadness, anxiety, and emotional dysregulation; and menopause-related psychological symptoms such as depressed mood, worry, and nervousness [15,16,17,18,19,20,21]. At the core of this phenomenon lies the bidirectional communication between the gut microbiome and the brain, known as the gut-brain axis, which has emerged as an important area of research in understanding mental well-being [22,23,24,25,26]. This relationship connecting intestinal bacteria, hormonal fluctuations, and psychological wellness is believed to occur through numerous complex interconnected biological processes within the gut-brain communication pathway [24,27,28,29]. Based on these mechanisms, emerging evidence suggests that modulating the gut microbiome may offer a novel therapeutic approach for various mental health conditions [22,23,27,30,31]. Therapeutic gut microbiome-targeted interventions include probiotics, which are live microorganisms that provide health benefits; prebiotics, which are non-digestible compounds primarily consisting of certain fibers that stimulate the growth and activity of beneficial gut bacteria; and synbiotics, which combine both probiotic and prebiotic components [32,33]. Additionally, paraprobiotics (or parabiotics) refer to inactivated microbial cells or their components that provide health benefits similar to live probiotics; postbiotics are the metabolic byproducts and secreted substances of probiotics; and psychobiotics are probiotics or prebiotics that exert beneficial effects on mental health [34,35,36].

Looking at the premenstrual phase, recent research has linked the gut microbiota to PMS, with growing evidence suggesting its influence on estrogen metabolism [37,38]. During this period, gut-targeted interventions such as probiotic supplementation may help alleviate PMS symptoms by modulating hormone levels, supporting the production of neurotransmitters such as serotonin and gamma-aminobutyric acid (GABA), and reducing inflammation [20,39].

During the perinatal period, women experience profound physiological and emotional changes related to hormonal fluctuations, which heighten the risk of mood disorders [18,40]. These disturbances can negatively impact neonatal outcomes, reduce breastfeeding rates, and influence long-term child development [40,41]. However, safety concerns regarding medication use during pregnancy and lactation often lead women to avoid pharmacological treatments [18]. Therefore, probiotics have emerged as a promising alternative, with clinical trials demonstrating their potential to mitigate perinatal and postnatal depression and anxiety [17,18,42].

As women approach menopause, typically around age fifty, they experience a natural decline in estrogen levels [43]. This major hormonal shift is associated with an increase in physical and psychological symptoms such as hot flashes, sleep disturbances, and anxiety, as well as higher risk of depression [44]. During this transition, evidence suggests that targeting the intestinal flora through probiotics and other gut-targeted approaches may effectively alleviate these menopausal symptoms while promoting better emotional and physical well-being [16,19,45,46,47].

Despite a growing body of primary research exploring how gut-targeted interventions may influence psychological wellness, a comprehensive synthesis of their efficacy specifically in women across key hormonal life stages (premenstrual, perinatal, and menopausal) is currently lacking. Our review is distinct from previous studies in that it exclusively and comprehensively synthesizes RCT evidence across these three hormonal life stages, addressing a critical knowledge gap in women’s health. Therefore, this systematic review and meta-analysis aimed to address this knowledge gap by systematically synthesizing evidence from RCTs on the efficacy of gut microbiome-targeted interventions in improving women’s mental health symptoms during these hormonal transitions.

## 2. Methods

This systematic review and meta-analysis used the Preferred Reporting Items for Systematic Reviews and Meta-Analyses (PRISMA) 2020 guidelines (checklist available in Supplementary File S1: Table S1). The study protocol was registered in the International Prospective Register of Systematic Reviews PROSPERO (registration number CRD420251089627).

### 2.1. Eligibility Criteria

Eligibility criteria for this review were established using the PICOS framework (population, intervention, comparison, outcome, study design) (Table 1). Studies published in English or French (based on the authors’ language proficiency) were included if they involved healthy women aged 18–65 years in one of three hormonal life stages: (1) women of reproductive age, including those in the premenstrual period or experiencing premenstrual syndrome or menstrual-related symptoms; (2) pregnant women or those up to 12 months postpartum; and (3) women experiencing menopausal symptoms during perimenopause or postmenopause. Exclusion criteria comprised studies including men or mixed-gender samples without sex-specific analyses, as well as participants with major psychiatric disorders requiring medication or with medical conditions known to affect the gut microbiome, such as inflammatory bowel disease, diabetes, or obesity. Eligible interventions encompassed probiotics, prebiotics, synbiotics, psychobiotics, paraprobiotics, postbiotics, and fermented foods with verified probiotic content; studies were excluded if these interventions were administered in combination with pharmacological or other treatments whose independent effects could not be determined, or if they involved antibiotics or other gut-depleting regimens. Acceptable comparators included placebo, no intervention, or standard care. The primary outcomes of interest were changes from baseline in validated measures of depression and anxiety symptoms, while secondary outcomes included other aspects of mental health (e.g., irritability, mood swings, stress) when available. Only RCTs were eligible for inclusion, with unpublished studies, pilot trials, and non-RCT designs excluded.

### 2.2. Search Strategy

A systematic search was conducted across the following electronic databases and registries following recommendations from a university librarian specialized in health sciences, covering the period from inception to August 2025: Embase, MEDLINE (PubMed), Web of Science, PsycINFO, CINAHL, Scopus, Food Science and Technology Abstracts (FSTA), Cochrane Central Register of Controlled Trials (CENTRAL), WHO International Clinical Trials Registry Platform, and ClinicalTrials.gov. The following keywords, along with relevant Medical Subject Headings (MeSH) were used, and key terms were “exploded” in databases when possible to broaden the search: (probiotics OR prebiotics OR synbiotics OR psychobiotics OR postbiotics OR paraprobiotics OR parabiotics OR fermented foods OR yogurt OR yoghurt OR kimchi OR kombucha OR kefir OR sauerkraut OR fermented milk products OR cultured milk products OR microbiome OR microbiota OR gut flora OR intestin* flora OR gut-brain axis OR gut dysbiosis) AND (premenstrual OR menstrua* OR premenstrual disorders OR premenstrual syndrome OR premenstrual symptoms OR PMS OR dysmenorrhea OR luteal phase OR follicular phase OR menarche OR perinatal* OR pregnancy OR postpartum OR PPD OR PND OR prenatal* OR postnatal* OR antenatal* OR menopaus* OR perimenopause*) AND (mental health OR mental well-being OR mental wellbeing OR psychological well-being OR psychological wellbeing OR depression OR anxiety OR emotional wellbeing OR emotional well-being OR psychological distress OR mood* OR stress) AND (randomized controlled trial OR randomised controlled trial OR RCT) (See Supplementary File S2 for an example of full electronic search strategy). Additionally, a citation check was performed by manually searching the reference lists of relevant studies to identify any other potentially eligible publications. No publication date restrictions were applied. All references were imported into Covidence software “www.covidence.org (accessed on 5 August 2025)” for the screening process. Duplicates were removed, and two reviewers independently evaluated study eligibility by screening titles and abstracts, followed by full-text review (N.D. and C.V.). Any disagreement was resolved through discussion.

### 2.3. Methodological Quality Appraisal

The methodological quality of the included studies was assessed using the Cochrane risk-of-bias tool for randomized trials (RoB 2.0) [48]. This tool evaluates six domains: (1) bias arising from the randomization process; (2) bias due to deviations from intended interventions; (3) bias due to missing outcome data; (4) bias in measurement of outcome; (5) bias in selection of the reported results; and (6) overall risk of bias. For each domain, studies were graded as “low risk of bias”, “some concerns”, or “high risk of bias”. Two reviewers independently assessed each study (N.D. and C.V.), and disagreements were resolved through discussion.

### 2.4. Data Extraction

Data extraction was performed independently by two reviewers (N.D. and C.V.), who systematically collected the following information from each included study: study name, country, study design, sample sizes of intervention and control groups, relevant subgroups if applicable, participant characteristics, mean age of participants in the treatment and control groups, relevant outcomes, intervention type, treatment duration, strain(s) or products used, daily dosage, delivery form, comparator, measurement scales, and mean changes from baseline with standard deviations for both intervention and control groups. Study authors were contacted when necessary to obtain additional information or clarification, which happened with two studies.

### 2.5. Statistical Analysis

Meta-analyses were conducted for the specified outcomes using a random-effects model to account for heterogeneity among studies. Comprehensive Meta-Analysis (CMA) version 4 [49] “https://meta-analysis.com (accessed on 10 August 2025)” was used for all statistical analyses. Effect sizes for continuous outcomes were calculated using standardized mean differences (SMD) with 95% confidence intervals (CI). The CMA software implements an inverse-variance weighting approach, whereby studies with more precise estimates contribute more heavily to the pooled effect. In the random-effects model, this weighting is adjusted to include both the sampling variance and the estimated between-study variance (τ^2^), allowing the model to account for heterogeneity across studies. Meta-regression analyses were performed to explore associations between treatment effects and intervention duration. Sensitivity analyses employed the one-study removal method to assess whether exclusion of any single trial significantly altered the overall effect size. Subgroup analyses by hormonal stage were planned but could not be conducted due to the limited number of studies within each subgroup. Statistical heterogeneity was examined using Cochran’s Q, the I^2^ statistic, and Tau^2^ (τ^2^). Cochran’s Q tests for the presence of heterogeneity, the I^2^ statistic reflects the proportion of total variance due to heterogeneity rather than chance (sampling error), and τ^2^ quantifies the absolute between-study variance in true effects [50]. Funnel plots and Egger’s test were planned to assess potential publication bias if at least ten studies were included in a meta-analysis [51]. However, this analysis was not conducted due to fewer than ten studies being included in the quantitative analysis [51].

## 3. Results

### 3.1. Study Selection

**Figure 1:**
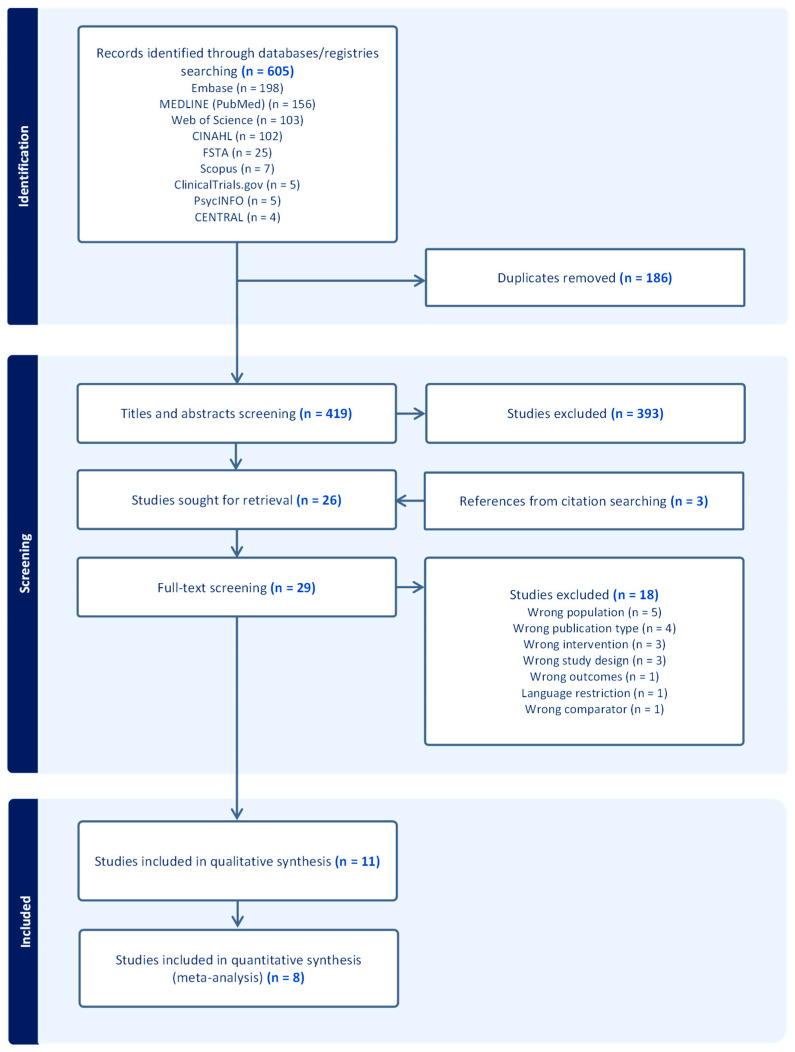
PRISMA flowchart.

A total of 605 records were identified through database and registry searches and imported into the Covidence software for screening. Following this, 186 duplicates were removed, and 419 articles were screened by title and abstract. Based on predefined inclusion and exclusion criteria, 393 studies were excluded. Subsequently, 29 studies, which included three from manual citation searches, underwent full-text screening. Of these, 18 studies were excluded for not meeting eligibility criteria (Supplementary File S1: Table S2). Finally, 11 studies were included in the qualitative synthesis, three of which were subsequently excluded from the quantitative analysis due to missing data, leaving eight studies for the meta-analysis. The PRISMA flowchart (Figure 1) outlines the study selection process and the reasons for excluding studies.

### 3.2. Study Characteristics

The 11 included studies [16,17,18,19,20,37,42,45,46,52,53] comprised nine double-blind and two triple-blind RCTs. All were published between 2017 and 2025 and written in English. Among these studies, four were conducted during the premenstrual hormonal stage [20,37,52,53], three during the perinatal period [17,18,42], and four during the menopausal period [16,19,45,46]. Although study authors were contacted when needed, three studies could not be included in the meta-analysis because they lacked either baseline data [17,42] or post-intervention data [37]. The eight studies included in the quantitative synthesis consisted of three in the premenstrual hormonal stage [20,52,53], one in the perinatal period [18], and four in the menopausal period [16,19,45,46]. Most included studies used probiotic supplements as treatment [17,18,19,20,42,45,52,53], two used paraprobiotics [37,46], and one used prebiotics [16]. All treatments were well tolerated with few adverse events reported. The study characteristics are shown in Table 2, and details about the interventions are provided in Table 3.

**Figure 2:**
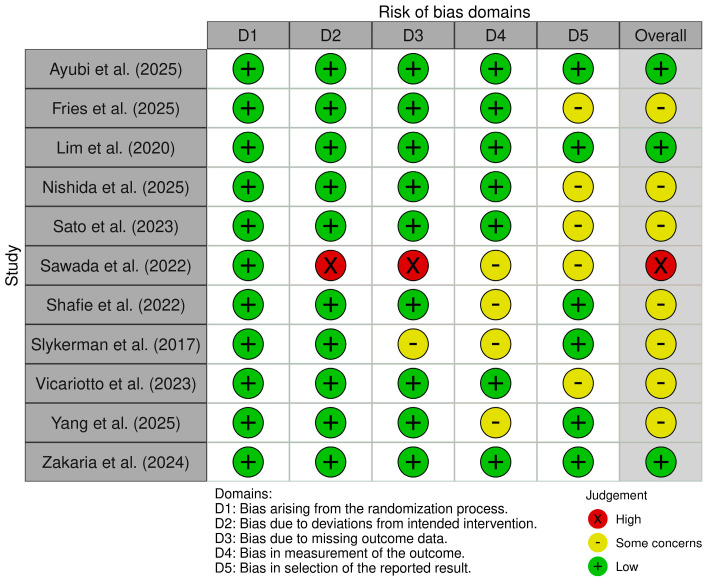
Quality assessment of included studies using the Cochrane’s risk-of-bias (RoB 2.0) [16,17,18,19,20,37,42,45,46,52,53].

According to Cochrane’s risk-of-bias tool for randomized trials (RoB 2.0) analysis, most included studies were graded as medium risk of bias overall (7/11, 64%), with 27% graded low risk (3/11), and 9% high risk (1/11) (Figure 2).

### 3.3. Effect of Gut-Targeted Interventions on Depression

**Figure 3:**
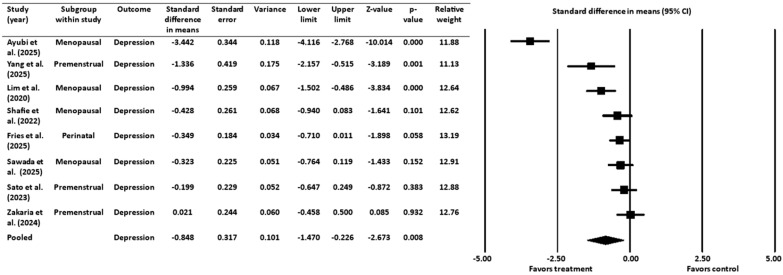
Effect of gut-targeted interventions on depression [16,18,19,20,45,46,52,53]. CI = confidence interval.

A meta-analysis of 8 RCTs [16,18,19,20,45,46,52,53] with a pooled sample size of 583 women across key hormonal stages of the female life cycle (premenstrual, perinatal/postpartum, and menopausal), assessed the efficacy of probiotics, paraprobiotics, or prebiotics in alleviating symptoms of depression. The standardized mean difference was used as the effect size index, and a random-effects model was employed (Figure 3). The results showed a statistically significant mean effect size of −0.848 with a 95% CI ranging from −1.470 to −0.226. The Z-value was −2.673 (*p* = 0.008), which allowed for rejection of the null hypothesis that the mean effect size equals zero. A negative effect size indicated that the treatment was favored over the control.

#### 3.3.1. Heterogeneity and Variation for Depression Outcome

The Q-statistic (Q = 87.102, degrees of freedom = 7, *p* ˂ 0.001) indicated significant heterogeneity among the studies. This finding was supported by an I^2^ value of 92%, suggesting that a large proportion of the observed variance was due to true effect size variation rather than sampling error. The analysis showed a between-study variance (τ^2^) of 0.729 with a standard deviation (τ) of 0.854, which provides an estimate of how much the true effect sizes vary across studies.

#### 3.3.2. Sensitivity Analysis for Depression Outcome

**Figure 4:**
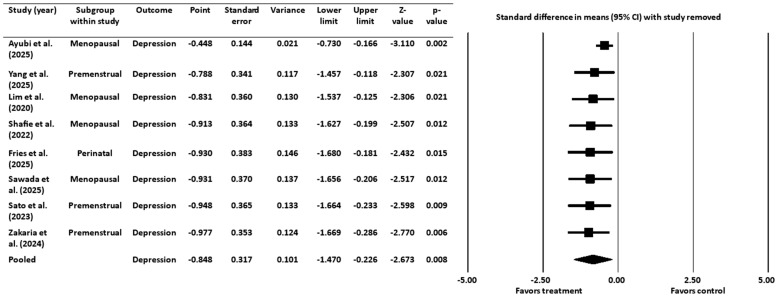
Sensitivity analysis for depression [16,18,19,20,45,46,52,53]. CI = confidence interval.

A sensitivity analysis was conducted to assess the robustness of the meta-analysis findings by systematically removing one study at a time and re-calculating the pooled effect size (Figure 4). The pooled effect size remained negative and statistically significant after the removal of each individual study, indicating that the overall findings were not dependent on any single study.

### 3.4. Effect of Gut-Targeted Interventions on Anxiety

**Figure 5:**
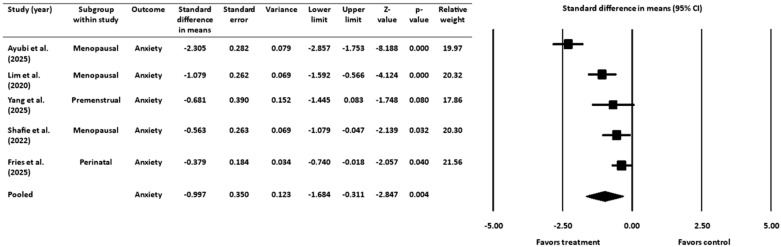
Effect of gut-targeted interventions on anxiety [16,18,19,45,52]. CI = confidence interval.

A meta-analysis was also performed on 5 RCTs [16,18,19,45,52], with a pooled sample size of 359 women, to examine the effect of probiotics and prebiotics on symptoms of anxiety during the premenstrual, perinatal/postpartum, or menopausal stages (Figure 5). The standardized difference in means was used as the effect size index, and a random-effects model was applied. The pooled analysis demonstrated a statistically significant mean effect size of −0.997, with a 95% CI ranging from −1.684 to −0.311. The Z-value was −2.847 (*p* = 0.004), allowing for the rejection of the null hypothesis that the mean effect size is zero. As with the depression analysis, the negative value of the effect size indicated that treatment groups experienced greater improvement in anxiety symptoms compared to control groups.

#### 3.4.1. Heterogeneity and Variation for Anxiety Outcome

The heterogeneity analysis revealed a Q-statistic of 35.310 (degrees of freedom = 4, *p* ˂ 0.001), indicating significant variability among the included studies. The I^2^ statistic was 89%, suggesting that most of the observed variance was due to actual differences in effect sizes rather than sampling error. The random-effects model estimated a between-study variance (τ^2^) of 0.535 and a standard deviation (τ) of 0.732.

#### 3.4.2. Sensitivity Analysis for Anxiety Outcome

**Figure 6:**
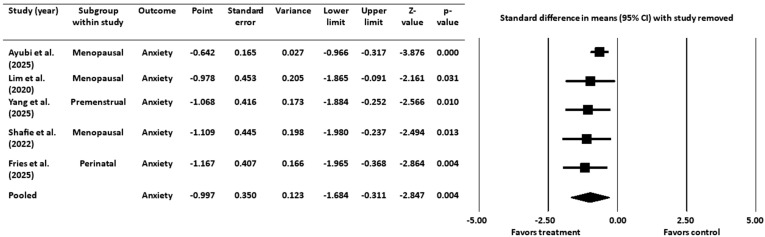
Sensitivity analysis for anxiety [16,18,19,45,52]. CI = confidence interval.

As with the depression analysis, a sensitivity analysis was conducted by sequentially removing each study and recalculating the pooled effect size (Figure 6). The negative effect size remained statistically significant in all cases, confirming that the findings were robust and not driven by any individual study.

### 3.5. Meta-Regression Analyses of Gut-Targeted Interventions on Duration of Treatment

Meta-regression analyses were conducted to explore whether the duration of treatment influenced its effectiveness for either depression or anxiety (Supplementary File S1: Table S3). For depression, the coefficient for treatment duration was non-significant (b = 0.0785, *p* = 0.1216). The model explained none of the observed heterogeneity in effect sizes (R2 analog = 0.00). For anxiety, the duration coefficient was also non-significant (b = 0.0621, *p* = 0.2784), accounting for only a small proportion of between-study variance (R^2^ analog = 0.03). These results indicate that the duration of treatment was not a significant predictor of treatment effectiveness for improving symptoms of depression or anxiety.

### 3.6. Additional Studies Excluded from the Quantitative Analyses

In addition to the studies included in the meta-analyses, three RCTs could not be included due to unavailable data, but nonetheless provide relevant insights. Nishida et al. (2021) [37] demonstrated that daily administration of the paraprobiotic *Lactobacillus gasseri* CP2305 over six menstrual cycles resulted in significant improvements in depression and anxiety symptoms associated with premenstrual symptoms compared to placebo controls. In the perinatal context, Slykerman and colleagues (2017) [17] found that women who received a probiotic supplement (*Lactobacillus rhamnosus* HN001) starting at 14–16 weeks of gestation through six months postpartum exhibited significantly lower rates of depression and anxiety symptoms relative to placebo. Similarly, Vicariotto et al. (2023) [42] reported significant improvements in maternal depression when participants were supplemented with the probiotics *Limosilactobacillus reuteri* PBS072 and *Bifidobacterium breve* BB077 during the first 90 days postpartum compared to controls.

### 3.7. Additional Mental Health Outcomes

Beyond depression and anxiety outcomes, some studies in this review examined additional mental health parameters, including irritability, mood swings, and stress. The findings for these secondary outcomes were mixed. Nishida et al. (2021) [37] found no statistically significant effect on irritability or mood swings in the context of premenstrual symptoms. However, both Sato et al. (2023) [20] and Yang et al. (2025) [52] reported statistically significant improvements in premenstrual irritability compared to placebo groups, although Sato et al. (2023) [20] observed no significant change in mood swings during this period. Finally, in the menopausal population, Ayubi et al. (2025) [19] reported that the intervention group showed statistically significant improvements in stress symptoms relative to placebo controls.

## 4. Discussion

### 4.1. Discussion of Main Findings

This systematic review and meta-analysis aimed to synthesize evidence from RCTs on the efficacy of gut microbiome-targeted interventions in improving mental health symptoms in women undergoing key hormonal transitions: the premenstrual phase, the perinatal/postpartum period, and menopause.

Our results from meta-analyses indicated that women who received interventions such as probiotics, prebiotics, or paraprobiotics, experienced a significant reduction in symptoms of depression (SMD = −0.848, *p* = 0.008) and anxiety (SMD = −0.997, *p* = 0.004) compared to control groups. The magnitude of these pooled effects suggests potential clinical relevance, being within or above the range commonly reported for established antidepressive pharmacological and psychological treatments [54,55,56,57,58].

For both depression and anxiety, the menopausal subgroup demonstrated the most robust and consistent evidence, although specific subgroup analyses by hormonal stage could not be conducted due to the limited number of studies within each subgroup. In the anxiety analysis, all three menopausal studies showed significant effects with particularly impressive results from Ayubi et al. (2025) [19] and Lim et al. (2020) [45], demonstrating large effect sizes (d = −2.305 and −1.079, respectively). The premenstrual subgroup exhibited different patterns across outcomes. Although the perinatal evidence is limited to a single study per outcome, the results showed promising effects, particularly for anxiety. This highlights a critical evidence gap given the high prevalence of perinatal mood and anxiety disorders and the limited safe treatment options available during this period.

Despite the overall positive findings, we observed substantial heterogeneity (I^2^ = 92% for depression, 89% for anxiety), indicating that intervention effects varied considerably across studies. The results from the analyses highlight this variability. For instance, in the depression meta-analysis, the Ayubi et al. (2025) [19] study on menopausal women showed a large beneficial effect (d = −3.442), while in contrast, the Zakaria et al. (2024) [53] study on perinatal women showed a non-significant effect size of d = 0.021. Similarly, for anxiety, the effect sizes ranged from a large negative effect in the Ayubi et al. (2025) [19] study on menopausal women (d = −2.305) to a modest, but still significant effect in the Fries et al. (2025) [18] study on perinatal women (d = −0.379). Therefore, this high level of heterogeneity suggests substantial variation in the true effect sizes across the included studies, which is not surprising given the diverse nature of the interventions, participant populations, and study designs. For example, the interventions varied widely in terms of type of treatment (probiotics, prebiotics, paraprobiotics), different bacterial strains and combinations, varying dosages, and treatment durations. Furthermore, the studies were conducted across three distinct hormonal life stages, each with its own unique physiological and hormonal landscape. These factors likely contributed to the observed variability in treatment effects. This diversity, however, also suggests that multiple approaches to gut microbiome intervention may be effective in providing beneficial mental health effects for these populations.

The sensitivity analyses confirmed that the overall findings were robust and not driven by any single study.

Interestingly, meta-regression analyses did not identify treatment duration as a significant moderator of effectiveness for either depression or anxiety outcomes, suggesting that factors other than intervention length may be more critical determinants of efficacy, although the small number of studies in the analyses limit the strength of this conclusion.

### 4.2. Comparison with Existing Literature

Our findings align with a growing body of evidence from several systematic reviews and meta-analyses suggesting promising therapeutic effects of gut-targeted interventions on mental health outcomes, although there are wide methodological variations across studies, including differences in treatment types, strains or products, dosage, duration, and study populations.

Among systematic reviews and meta-analyses on perinatal and postpartum mental health, Halemani et al. (2023) [59] reported that probiotic supplementation reduced depression and anxiety in both pregnant and lactating women. For their part, Desai et al. (2021) [60] found limited but promising evidence for the effectiveness of probiotics in reducing anxiety and depressive symptoms during pregnancy. Finally, an umbrella review by Alemu et al. (2024) [61] concluded that probiotics were beneficial in reducing anxiety symptoms during pregnancy and depression during lactation, but highlighted the need for more research in the perinatal period.

Research also suggests a useful role for these interventions during the menopausal transition. A systematic review and meta-analysis by Andrews et al. (2025) [62] found that probiotics improved menopausal symptoms, including significant improvements in psychological symptoms compared with placebo. Benefits were also observed for urogenital and bone health, indicating the potential of probiotics to alleviate a range of symptoms experienced during this life stage.

By contrast, evidence in the premenstrual period remains surprisingly scarce. Nevertheless, a cross-sectional study by Takeda et al. (2022) [63] discovered that decreased levels of the intestinal bacteria *Parabacteroides* and *Megasphaera* were associated with more severe premenstrual symptoms compared with controls. Additionally, a clinical study by Okuma et al. (2022) [64] identified distinct microbiota profiles in women with PMS, including markedly elevated *Collinsella* compared with the control group. Similarly, Yao et al. (2024) [65] reported a significant association between menstrual disorders and the intestinal bacteria *Escherichia/Shigella*. Notably, these bacteria were also found to be elevated in patients with anxiety and depression [66,67,68], underscoring the need for further research into microbial pathways that may influence menstruation-related mental health.

Among meta-analytic findings in more general populations, Zhang et al. (2023) [69] reported significant improvements in depressive symptoms among adults diagnosed with depression who were supplemented with prebiotics, probiotics, or synbiotics, with subgroup analyses indicating that probiotics were the main driver of these effects. In addition, Asad et al. (2025) [70] found that among clinically diagnosed adults with depression and/or anxiety, probiotics substantially reduced depression and moderately reduced anxiety compared with controls, while prebiotics showed only a nonsignificant trend. Similarly, a systematic review and meta-analysis by Moshfeghinia et al. (2025) [71] concluded that probiotics, prebiotics, or synbiotics significantly reduced depression and anxiety symptoms in patients with depression compared with controls, despite high heterogeneity across studies. Complementing these findings, a meta-analysis of cohort studies by Luo et al. (2023) [72] reported that consumption of fermented dairy foods was significantly associated with a reduced risk of depression. Finally, evidence from a meta-review of systematic reviews by Anguiano Morán et al. (2025) [73] confirmed that prebiotics, probiotics, and synbiotics tend to alleviate depressive symptoms in individuals with depression compared to controls. Nonetheless, they noted that evidence remains limited for certain age groups, such as children, adolescents, and older adults, and highlighted the need for further research on the effects of specific probiotic strains and treatment combinations.

Thus, taken together, recent clinical evidence suggests an encouraging overall trend supporting the mental health benefits of gut-brain axis interventions, consistent with our review findings, despite methodological differences and the emerging nature of clinical research in this field.

### 4.3. Potential Mechanisms

The gut-brain axis is a bidirectional communication pathway that connects the central nervous system (CNS) and the enteric nervous system (ENS), allowing the brain and gut to influence each other [74]. Communication occurs through four main pathways: vagal and spinal afferent neurons, cytokines, endocrine hormones, and microbial factors [75]. The gut microbiome, which includes diverse bacteria, produces essential metabolites, vitamins, and other compounds like lipopolysaccharides and peptides [75]. The microbiome is in close contact with gut endocrine cells, which it activates to produce several hormones [75]. Studies have shown that the microbiota can actually influence behavior, stress, emotion, pain response, and brain biochemistry [76]. Probiotics have also been found to affect brain function through the gut-brain axis [77]. Interestingly, a 4-week RCT conducted by Rode et al. (2022) on healthy adults found that a multi-strain probiotic intervention resulted in gray matter volume changes in the brain compared to placebo [78]. Furthermore, the probiotic intervention showed beneficial effects on depression and sleep patterns, as well as markers of gut-brain interactions, such as serum serotonin concentrations [78].

The gut microbiome’s composition and diversity vary between individuals and are shaped by diet, lifestyle, genetics, and medication use, including antibiotics [79]. Gut dysbiosis, defined as an imbalance in microbial composition or reduced diversity, has been associated with numerous conditions, including mental health disorders such as depression and anxiety [24]. Some probiotic strains can synthesize neurotransmitters involved in emotional balance and brain function, including GABA, acetylcholine, norepinephrine, dopamine, and serotonin [32,75,80,81,82]. Notably, up to 95% of the body’s serotonin, which plays an important role in mood regulation, is produced in the gut mucosa, and its secretion from enterochromaffin cells is modulated by microbial metabolites [83,84]. Another key mechanism linking the gut microbiome to depression involves tryptophan metabolism, as tryptophan serves as a precursor for serotonin [85,86]. Beneficial microbes can reduce host tryptophan degradation via the kynurenine pathway, thereby increasing its availability for serotonin synthesis [87,88]. In addition, certain bacteria are capable of directly producing tryptophan, whereas others can metabolize it through tryptophanase activity [85,89].

Hormonal fluctuations can also affect the gut microbiome [90]. Estrogen influences microbial composition, while certain bacteria, collectively termed the estrobolome, metabolize estrogens through β-glucuronidase activity, thereby affecting circulating estrogen levels [38,91]. Dysbiosis can lower estrogen availability by reducing its deconjugation [38,91]. Distinct microbiome profiles have been observed across the female lifespan [65,92]. The cyclical rise and fall of reproductive hormones such as estradiol and progesterone, combined with changes in pituitary hormones, prostaglandins, and neurotransmitters in the brain, are linked to the physiological and psychological symptoms experienced during the menstrual cycle [65,93]. Although data remain limited, cyclic gut microbiome alterations may contribute to premenstrual symptoms [28,65,93]. Alongside endocrine, metabolic, and immune changes, pregnancy is also associated with shifts in gut microbiota composition [94,95,96]. After parturition, the sharp decline in estradiol and progesterone may be influenced by microbiota-related enzymes (e.g., β-glucuronidase), potentially contributing to postpartum depression [97]. In premenopausal women, higher estrogen levels promote intestinal microbial diversity and inhibit the growth of harmful bacteria [92]. By contrast, menopause, which is characterized by estrogen decline and ovarian dysfunction, correlates with reduced microbial diversity and shifts in its composition [98,99,100]. During perimenopause, beneficial taxa such as *Lactobacillus* and *Bifidobacterium* decline, while potentially harmful bacteria like *Enterobacter* increase [99]. Notably, postmenopausal microbiomes have been found to resemble those of men, particularly in overall microbial diversity patterns [101].

Finally, inflammation represents another key link between gut health and mood disorders [22,102]. Dysregulated hypothalamic-pituitary-adrenal (HPA) axis activity, driven by pro-inflammatory cytokines, is a well-established biological marker of depression and anxiety [22,102]. Gastrointestinal inflammation induces pro-inflammatory cytokines such as tumor necrosis factor-alpha (TNF-α) and interleukin-6 (IL-6) [103,104]. These cytokines can influence brain function and mood through immune-to-brain communication pathways, including signaling across the blood-brain barrier, activation of endothelial and glial cells, and vagal nerve stimulation [22,105,106]. Elevated IL-6 and TNF-α levels are frequently associated with depressive and anxiety symptoms [105,107]. Several RCTs have reported that probiotics can mitigate inflammation and improve mental health outcomes. For example, an 8-week RCT by Lee et al. (2021) [108] tested a probiotic mixture in adults and observed that the treated group had a significant reduction in depressive symptoms compared to placebo. The authors also reported a significant reduction in serum pro-inflammatory cytokine IL-6 levels, as well as beneficial changes in the gut microbiota composition in the probiotic group [108]. Similarly, Lew et al. (2019) [109] conducted a 12-week RCT in adults, finding that probiotic supplementation with *Lactobacillus plantarum* P8 provided beneficial effects in reducing anxiety compared to placebo. Participants in the treatment group also exhibited lower plasma levels of pro-inflammatory cytokines, including interferon-gamma (IFN-γ) and TNF-α, along with improved cognitive and memory performance relative to placebo [109].

### 4.4. Limitations

Several limitations of this review must be acknowledged. The primary limitation concerns the substantial heterogeneity across studies, reflecting the varying nature of interventions, methodologies, and population types. While this is not uncommon in meta-analyses of diverse interventions, it limits the ability to provide specific clinical recommendations regarding optimal bacterial strains, dosages, or treatment protocols.

The small number of eligible RCTs in the meta-analyses, particularly the single study available for the perinatal subgroup, means that our conclusions for this specific population are based on limited data and should be interpreted with caution. Moreover, the wide confidence intervals around the pooled estimates indicate uncertainty in the true magnitude of the effects and suggest that these findings should be interpreted with caution. Additionally, subgroup analysis by hormonal life stage could not be performed due to the small sample size, which limits our ability to determine whether the efficacy of the interventions differs significantly between the three hormonal periods. We conducted a meta-regression on treatment duration for both depression and anxiety outcomes; however, further meta-regressions were not feasible due to the substantial heterogeneity in intervention types (different probiotic/paraprobiotic strains and a single prebiotic product) and the lack of a common dosage scale across studies, which prevented direct comparison of dosage levels. These factors restricted our ability to meaningfully model additional moderators.

Another limitation is that most included studies relied on self-reported measures of depression and anxiety, assessed with different measuring tools. Although validated, they remain subject to reporting bias and contribute to heterogeneity when results are compared across scales. Furthermore, the populations included in this review primarily comprised healthy subjects; therefore, the findings may not be generalizable to all women, particularly those with severe symptoms requiring medical intervention or with comorbid diseases. Additionally, despite the randomized design of the included studies, important influences such as diet, lifestyle, body weight, and baseline gut microbiome characteristics in participants were not consistently controlled or measured in the studies. This leaves open the possibility of residual confounding or effect modification in the observed relationship between gut microbiome interventions and mental health outcomes. Finally, assessment of publication bias was not possible due to the small number of studies (˂10), and the potential for selective reporting of positive outcomes cannot be ruled out.

### 4.5. Future Directions

The gut microbiome is emerging as a promising target for advancing women’s mental health, yet research in this area remains limited despite heightened risk of psychological distress associated with key hormonal transitions such as the premenstrual, perinatal/postpartum, and menopausal periods. Addressing this gap is critical, and future research should prioritize the development of gut-targeted therapies that account for individual hormonal, metabolic, and microbiome characteristics, while also mapping how hormonal fluctuations across the menstrual cycle, pregnancy, and menopause interact with intestinal microbial composition through the gut-brain axis to shape mental health outcomes. Further research is also needed to determine optimal intervention types, microbial strains and combinations, dosing regimens, and timing strategies for specific psychological outcomes during these periods.

To enhance clinical impact, trials should incorporate standardized microbiome and biomarker assessments, including measures of hormonal status and markers of stress and inflammation, while accounting for moderators such as comorbidities, medications, diet, lifestyle, and sleep. Larger and more diverse cohorts are needed to ensure findings are generalizable, with particular attention to underrepresented populations, such as in the premenstrual period, where evidence is especially limited. Comparative effectiveness research should evaluate how gut-targeted strategies perform relative to established psychiatric treatments and clarify their potential as preventive or adjunctive options during high-risk hormonal transitions. Equally important are long-term follow-up studies to determine whether therapeutic benefits persist after discontinuation of supplementation, to inform clinical guidelines and recommendations.

## 5. Conclusions

This systematic review and meta-analysis provide encouraging evidence that gut microbiome-targeted interventions can effectively reduce symptoms of depression and anxiety in women during key hormonal stages, including the premenstrual, perinatal/postpartum, and menopausal periods. The meta-analyses of RCTs showed a statistically significant reduction in depression symptoms (SMD = −0.848, *p* = 0.008) and anxiety symptoms (SMD = −0.997, *p* = 0.004) in women with the use of gut-targeted interventions (probiotics, prebiotics, or paraprobiotics).

Despite high heterogeneity among studies, our sensitivity analyses confirmed the robustness of these findings, indicating that no single study drove the overall effect. This suggests that various intervention approaches may offer therapeutic benefits. While our meta-regression analyses did not identify treatment duration as a significant moderator, more research is needed to explore additional moderators and to identify predictive biomarkers such as gut microbiome characteristics, inflammatory profiles, and hormone levels, which will be needed for developing personalized therapeutic strategies.

Overall, these findings highlight the potential of gut-targeted interventions as a generally well-tolerated and promising complementary therapeutic approach, while also underscoring the need for further research to clarify the specific pathways through which the intestinal microbiome influences the gut-brain axis in these populations. Future studies should focus on identifying optimal treatment protocols for gut-targeted interventions to support the development of clear clinical guidelines and to maximize therapeutic benefits for women’s mental health during these hormonal transitions characterized by heightened vulnerability to mood and anxiety disorders.

## Figures and Tables

**Table 1:** PICOS strategy description.

Parameter	Description
Population (P)	Inclusion criteria: Healthy women aged 18–65 years. Participants in one of three hormonal life stages: 1. Premenstrual group: women of reproductive age experiencing premenstrual syndrome or menstrual-related symptoms. 2. Perinatal group: pregnant women or women up to 12 months postpartum. 3. Menopausal group: women experiencing menopausal symptoms during perimenopause or postmenopause. Exclusion criteria: Men or mixed-gender studies without sex-specific data. Women with major psychiatric disorders requiring medication. Women with comorbid conditions affecting gut microbiome (e.g., inflammatory bowel disease, diabetes, obesity).
Intervention (I)	Inclusion criteria: Probiotics, prebiotics, synbiotics, psychobiotics, paraprobiotics, postbiotics, or fermented foods with documented probiotic content. Exclusion criteria: Interventions combined with other treatments (e.g., pharmacological) where effects cannot be separated. Interventions involving antibiotics or other gut-depleting interventions.
Comparison (C)	Placebo, no intervention, standard care.
Outcome (O)	Primary outcomes: changes in scores on validated scales measuring symptoms of depression and anxiety. Secondary outcomes: changes in other mental health-related outcomes.
Study design (S)	Inclusion criteria: Randomized controlled trials (RCTs) Exclusion criteria: Unpublished trials, pilot studies, non-RCT designs.

**Table 2:** Characteristics of the included studies (n = 11).

Study, Country	Hormonal Life Stage Group	Specific Period Covered	Study Design	Participants	Average Age (T, C)	Sample Size (T, C)	Intervention Type	Comparator	Duration of Treatment (Weeks)	Mental Health Outcome(s) Measured	Measurement Tool(s)
Nishida et al. (2021) [37] Japan	Premenstrual	N/A	Double-blind RCT	Female students	T = ~21; C = ~22	T = 25; C = 31	Paraprobiotics	Placebo	~26	Depression, Anxiety, Irritability, Mood swings	PMTS-VAS
Sato et al. (2023) [20] Japan	Premenstrual	N/A	Double-blind RCT	Women with menstrual-related symptoms	T = ~32; C = ~32	T = 39; C = 38	Probiotics	Placebo	~12	Depression, Irritability, Mood swings	MDQ, VAS
Yang et al. (2025) [52] Taiwan	Premenstrual	N/A	Double-blind RCT	Women with primary dysmenorrhea	T = ~22; C = ~25	T = 15; C = 13	Probiotics	Placebo	10	Depression, Anxiety, Irritability	PSST
Zakaria et al. (2024) [53] Malaysia	Premenstrual	N/A	Double-blind RCT	Women with primary dysmenorrhea	T = ~25; C = ~26	T = 34; C = 33	Probiotics	Placebo	12	Depression	SF12v2
Fries et al. (2025) [18] China	Perinatal	Pregnancy and Postpartum	Double-blind RCT	Pregnant women	T = ~32; C = ~32	T = 61; C = 59	Probiotics	Placebo	~22	Depression, Anxiety	EPDS, STAI-S
Slykerman et al. (2017) [17] New Zealand	Perinatal	Pregnancy and postpartum	Double-blind RCT	Pregnant women	T = ~34; C = ~34	Depression: T = 194; C = 187 Anxiety: T = 192; C = 187	Probiotics	Placebo	~52	Depression, Anxiety	EPDS, STAI6
Vicariotto et al. (2023) [42] Italy	Perinatal	Postpartum	Double-blind RCT	Women in their first trimester postpartum	T = ~33; C = ~33	T = 95; C = 95	Probiotics	Placebo	~13	Depression	EPDS
Ayubi et al. (2025) [19] Iran	Menopausal	Postmenopause	Triple-blind RCT	Menopausal women	T = ~51; C = ~51	T = 42; C = 42	Probiotics	Placebo	6	Depression, Anxiety, Stress	DASS21
Lim et al. (2020) [45] Korea	Menopausal	Postmenopause	Double-blind RCT	Menopausal women	T = ~54; C = ~52	T = 32; C = 35	Probiotics	Placebo	12	Depression, Anxiety	KMI
Sawada et al. (2022) [46] Japan	Menopausal	Perimenopause	Double-blind RCT	Perimenopausal women	T = ~46; C = ~45	T = 40; C = 40	Paraprobiotics	Placebo	~24	Depression	GCS
Shafie et al. (2022) [16] Iran	Menopausal	Postmenopause	Triple-blind RCT	Menopausal women	T = ~52; C = ~52	T = 30; C = 30	Prebiotics	Placebo	6	Depression, Anxiety	GCS

C = control group; DASS21 = 21-item depression anxiety and stress scale; EPDS = Edinburgh postnatal depression scale; GCS = Greene climacteric scale; KMI = Kupperman menopausal index; MDQ = menstrual distress questionnaire; n = sample size; N/A = not applicable; T = treatment group; PMTS-VAS = premenstrual tension syndrome-visual analog scale; PSST = premenstrual symptoms screening tool; RCT = randomized controlled trial; SF12v2 = short-form 12-item version 2; STAI-S = state-trait anxiety inventory state subscale; STAI6 = state trait anxiety inventory 6 item version; VAS = visual analog scale.

**Table 3:** Details about gut-targeted interventions of included studies (n = 11).

Study	Group	Intervention Type	Strain(s)/Product	Daily Dosage	Delivery Form
Nishida et al. (2021) [37]	Premenstrual	Paraprobiotics	*Lactobacillus gasseri* CP2305	1 × 10^10^ BC	Tablet
Sato et al. (2023) [20]	Premenstrual	Probiotics	*Lactobacillus paragasseri* OLL2809	1 × 10^10^ BC	Tablet
Yang et al. (2025) [52]	Premenstrual	Probiotics	*Bifidobacterium longum subsp. longum* OLP-01, *L. plantarum* PL-02, and *Lactococcus lactis* LY-66	1.5 × 10^10^ CFU	Capsule
Zakaria et al. (2024) [53]	Premenstrual	Probiotics	*Lactobacillus acidophilus* BCMC 12130, *Lactobacillus casei* subsp BCMC 12313, *Lactobacillus lactis* BCMC 12451, *Bifidobacterium bifidum* BCMC 02290, *Bifidobacterium longum* BCMC 02120, and *Bifidobacterium infantis* BCMC 02129	1 × 10^10^ CFU	Sachet
Fries et al. (2025) [18]	Perinatal	Probiotics	*Bifidobacterium longum* NCC3001	1 × 10^10^ CFU	Sachet
Slykerman et al. (2017) [17]	Perinatal	Probiotics	*Lactobacillus rhamnosus* HN001	6 × 10^9^ CFU	Capsule
Vicariotto et al. (2023) [42]	Perinatal	Probiotics	*Limosilactobacillus reuteri* PBS072 and *Bifidobacterium breve* BB077	4 × 10^9^ CFU	Capsule
Ayubi et al. (2025) [19]	Menopausal	Probiotics	*Lactobacillus plantarum, Lactobacillus casei, L. acidophilus, L. bulgaricus, Bifidobacterium infantis, Bifidobacterium longum, Bifidobacterium breve*, and *S. thermophilus*	4.5 × 10^11^ CFU	Capsule
Lim et al. (2020) [45]	Menopausal	Probiotics	*Lactobacillus acidophilus* YT1	1 × 10^8^ CFU	Sachet
Sawada et al. (2022) [46]	Menopausal	Paraprobiotics	*Lactobacillus gasseri* CP2305	1 × 10^10^ BC	Tablet
Shafie et al. (2022) [16]	Menopausal	Prebiotics	Long-chain Inulin	1.5 g	Yogurt

BC = bacteria cells; BCMC = B-Crobes Microbial Cells; CFU = colony-forming units; g = grams; n = sample size.

## Data Availability

Data sharing is not applicable to this article.

## Supplementary Materials

The following supporting information can be downloaded at: https://www.mdpi.com/article/10.3390/healthcare13222851/s1, Supplementary File S1: Table S1: PRISMA 2020 Checklist; Table S2: Studies excluded after full-text screening (n = 18); Table S3: Results of meta-regressions on duration of treatment (weeks) for depression and anxiety (random-effects). Supplementary File S2: Example of full electronic search strategy.

## Abbreviations

CI	Confidence interval
CMA	Comprehensive Meta-Analysis
CNS	Central nervous system
ENS	Enteric nervous system
FSTA	Food Science and Technology Abstract
GABA	Gamma-aminobutyric acid
HPA	Hypothalamic–pituitary–adrenal
IFN-γ	Interferon-gamma
IL-6	Interleukin-6
MeSH	Medical subject headings
PMS	premenstrual syndrome
PND	Perinatal depression
PPD	Postpartum depression
PRISMA	Preferred Reporting Items for Systematic Reviews and Meta-Analysis
RCT	Randomized controlled trial
SMD	Standardized mean difference
TNF-α	Tumor necrosis factor-alpha
WHO	World Health Organization
