# Supplementary material for: Efficacy of Gut Microbiome-Targeted Interventions on Mental Health Symptoms in Women Across Key Hormonal Life Stages: A Systematic Review and Meta-Analysis of Randomized Controlled Trials
Source: Healthcare (Basel). 2025 Nov 10;13(22):2851. doi: 10.3390/healthcare13222851 (PMC12652156; doi:10.3390/healthcare13222851)
Supplement: Supplementary file 1 [file healthcare-13-02851-s001.zip › healthcare-3934528-Supplementary File 1.pdf]

Supplementary Materials

Table S1. PRISMA 2020 Checklist

Section and Topic	Item #	Checklist item	Location where item is reported
**TITLE**			
Title	1	Identify the report as a systematic review.	Title
**ABSTRACT**			
Abstract	2	See the PRISMA 2020 for Abstracts checklist.	Abstract
**INTRODUCTION**			
Rationale	3	Describe the rationale for the review in the context of existing knowledge.	Introduction
Objectives	4	Provide an explicit statement of the objective(s) or question(s) the review addresses.	Introduction
**METHODS**			
Eligibility criteria	5	Specify the inclusion and exclusion criteria for the review and how studies were grouped for the syntheses.	Methods, Table 1
Information sources	6	Specify all databases, registers, websites, organisations, reference lists and other sources searched or consulted to identify studies. Specify the date when each source was last searched or consulted.	Methods
Search strategy	7	Present the full search strategies for all databases, registers and websites, including any filters and limits used.	Methods
Selection process	8	Specify the methods used to decide whether a study met the inclusion criteria of the review, including how many reviewers screened each record and each report retrieved, whether they worked independently, and if applicable, details of automation tools used in the process.	Methods
Data collection process	9	Specify the methods used to collect data from reports, including how many reviewers collected data from each report, whether they worked independently, any processes for obtaining or confirming data from study investigators, and if applicable, details of automation tools used in the process.	Methods
Data items	10a	List and define all outcomes for which data were sought. Specify whether all results that were compatible with each outcome domain in each study were sought (e.g. for all measures, time points, analyses), and if not, the methods used to decide which results to collect.	Methods, Table 1
	10b	List and define all other variables for which data were sought (e.g. participant and intervention characteristics, funding sources). Describe any assumptions made about any missing or unclear information.	Methods
Study risk of bias assessment	11	Specify the methods used to assess risk of bias in the included studies, including details of the tool(s) used, how many reviewers assessed each study and whether they worked independently, and if applicable, details of automation tools used in the process.	Methods
Effect measures	12	Specify for each outcome the effect measure(s) (e.g. risk ratio, mean difference) used in the synthesis or presentation of results.	Methods
Synthesis methods	13a	Describe the processes used to decide which studies were eligible for each synthesis (e.g. tabulating the study intervention characteristics and comparing against the planned groups for each synthesis (item #5)).	Methods
	13b	Describe any methods required to prepare the data for presentation or synthesis, such as handling of missing summary statistics, or data conversions.	Methods
	13c	Describe any methods used to tabulate or visually display results of individual studies and syntheses.	Methods
	13d	Describe any methods used to synthesize results and provide a rationale for the choice(s). If meta-analysis was performed, describe the model(s), method(s) to identify the presence and extent of statistical heterogeneity, and software package(s) used.	Methods
	13e	Describe any methods used to explore possible causes of heterogeneity among study results (e.g. subgroup analysis, meta-regression).	Methods
	13f	Describe any sensitivity analyses conducted to assess robustness of the synthesized results.	Methods

Section and Topic	Item #	Checklist item	Location where item is reported
Reporting bias assessment	14	Describe any methods used to assess risk of bias due to missing results in a synthesis (arising from reporting biases).	Methods
Certainty assessment	15	Describe any methods used to assess certainty (or confidence) in the body of evidence for an outcome.	Methods
**RESULTS**			
Study selection	16a	Describe the results of the search and selection process, from the number of records identified in the search to the number of studies included in the review, ideally using a flow diagram.	Results, Figure 1
	16b	Cite studies that might appear to meet the inclusion criteria, but which were excluded, and explain why they were excluded.	Results, Table S2
Study characteristics	17	Cite each included study and present its characteristics.	Results, Tables 2, 3
Risk of bias in studies	18	Present assessments of risk of bias for each included study.	Results, Figure 2
Results of individual studies	19	For all outcomes, present, for each study: (a) summary statistics for each group (where appropriate) and (b) an effect estimate and its precision (e.g. confidence/credible interval), ideally using structured tables or plots.	Results, Figures 3, 5
Results of syntheses	20a	For each synthesis, briefly summarise the characteristics and risk of bias among contributing studies.	Results, Figure 2
	20b	Present results of all statistical syntheses conducted. If meta-analysis was done, present for each the summary estimate and its precision (e.g. confidence/credible interval) and measures of statistical heterogeneity. If comparing groups, describe the direction of the effect.	Results, Figures 3, 5, Table S3
	20c	Present results of all investigations of possible causes of heterogeneity among study results.	Results
	20d	Present results of all sensitivity analyses conducted to assess the robustness of the synthesized results.	Results, Figures 4, 6
Reporting biases	21	Present assessments of risk of bias due to missing results (arising from reporting biases) for each synthesis assessed.	Results, Figure 2
Certainty of evidence	22	Present assessments of certainty (or confidence) in the body of evidence for each outcome assessed.	Results, Figure 2
**DISCUSSION**			
Discussion	23a	Provide a general interpretation of the results in the context of other evidence.	Discussion
	23b	Discuss any limitations of the evidence included in the review.	Discussion
	23c	Discuss any limitations of the review processes used.	Discussion
	23d	Discuss implications of the results for practice, policy, and future research.	Discussion
**OTHER INFORMATION**			
Registration and protocol	24a	Provide registration information for the review, including register name and registration number, or state that the review was not registered.	Methods
	24b	Indicate where the review protocol can be accessed, or state that a protocol was not prepared.	Methods
	24c	Describe and explain any amendments to information provided at registration or in the protocol.	Methods
Support	25	Describe sources of financial or non-financial support for the review, and the role of the funders or sponsors in the review.	Funding
Competing	26	Declare any competing interests of review authors.	Conflicts of

Section and Topic	Item #	Checklist item	Location where item is reported
interests			Interest
Availability of data, code and other materials	27	Report which of the following are publicly available and where they can be found: template data collection forms; data extracted from included studies; data used for all analyses; analytic code; any other materials used in the review.	Results, Tables 2, 3

Table S2. Studies excluded after full-text screening (n = 18)

	Reference	Reasons for exclusion
1.	Al Kassaa, I., & Fuad, M. (2024). Effects of lacticaseibacillus rhamnosus HN001 on happiness and mental well-being: Findings from a randomized controlled trial. NUTRIENTS, 16(17). https://doi.org/10.3390/nu16172936	Wrong population
2.	Asemi, Z., Jazayeri, S., Najafi, M., Samimi, M., Mofid, V., Shidfar, F., Shakeri, H., & Esmaillzadeh, A. (2012). Effect of daily consumption of probiotic yogurt on oxidative stress in pregnant women: A randomized controlled clinical trial. Annals of Nutrition and Metabolism, 60(1), 62–68. https://doi.org/10.1159/000335468	Wrong outcomes
3.	Bozdoğan, F. B. K., Kabaran, S., & Tazeoğlu, A. (2024). Effect of probiotic supplementation on maternal depression, anxiety and attachment in gestational diabetes by improving mediterranean diet quality: A randomized controlled trial. Clinical and Experimental Obstetrics & Gynecology, 51(11), Article 11. https://doi.org/10.31083/j.ceog5111237	Wrong population
4.	Browne, P. D., Bolte, A. C., Besseling-van der Vaart, I., Claassen, E., & de Weerth, C. (2021). Probiotics as a treatment for prenatal maternal anxiety and depression: A double-blind randomized pilot trial. Scientific Reports, 11(1), 3051. https://doi.org/10.1038/s41598-021-81204-9	Wrong study design
5.	Chung Shan Medical University. (2022). To evaluate the improvement effect of supplementing hericium erinaceus mycelium capsules and grape king probiotic capsules of the grape king bio on physiological and psychological symptoms of premenstrual syndrome. *Clinicaltrials.Gov*.	Study not published yet
6.	Dawe, J. P., McCowan, L. M. E., Wilson, J., Okesene-Gafa, K. A. M., & Serlachius, A. S. (2020). Probiotics and maternal mental health: A randomised controlled trial among pregnant women with obesity. Scientific Reports, 10(1), 1291. https://doi.org/10.1038/s41598-020-58129-w	Wrong population
7.	El-Heis, S., Barton, S. J., Chang, H. F., Nield, H., Cox, V., Galani, S., Cutfield, W., Chan, S.-Y., & Godfrey, K. M. (2024). Maternal mood, anxiety and mental health functioning after combined myo-inositol, probiotics, micronutrient supplementation from preconception: Findings from the NiPPeR RCT. Psychiatry Research, 334, 115813. https://doi.org/10.1016/j.psychres.2024.115813	Wrong intervention
8.	Hulkkonen P, Kataja EL, Vahlberg T, Koivuniemi E, Houttu N, Pellonperä O, Mokkala K, Karlsson H, & Laitinen K. (2021). The efficacy of probiotics and/or n-3 long-chain polyunsaturated fatty acids intervention on maternal prenatal and postnatal depressive and anxiety symptoms among overweight and obese women. J Affect Disord, 289, 21–30. https://doi.org/10.1016/j.jad.2021.04.006	Wrong population
9.	Judkins, T. C., Blaufus, D., Taft, D., Oula, M.-L., Binda, S., & Langkamp-Henken, B. (2025). The effect of a probiotic on gastrointestinal symptoms due to menstruation in healthy females on oral contraceptives: A randomized, double-blind, placebo-controlled trial. Current Developments in Nutrition, 9. https://doi.org/10.1016/j.cdnut.2025.107144	Full article unavailable
10.	Liu, A. T., Chen, S., Jena, P. K., Sheng, L., Hu, Y., & Wan, Y.-J. Y. (2021). Probiotics improve gastrointestinal function and life quality in pregnancy. Nutrients, 13(11), 3931. https://doi.org/10.3390/nu13113931	Wrong study design
11.	Nisaa A.A., Mageswary U., Pei X., Kadir M.N., Oon C.E., Rajendran D., Tan J.J., Roslan F.F., Balasubramaniam S.D., Sany S., Ismail E.H.E., Azizan A.S., & Liong M.T. (2025). Probiotic enhanced immunity and mental wellbeing of generally healthy women: A randomised, placebo-controlled and double-blind study. Beneficial Microbes, (Nisaa, Mageswary, Liong) School of Industrial Technology, Universiti Sains Malaysia, Penang, Malaysia(Pei) College of Culinary and Food Science Engineering, Sichuan Tourism University, Chengdu, China(Kadir, Ismail) Obstetrics and Gynaecology, Hospital Un, 1EP – 18. https://doi.org/10.1163/18762891-bja00061	Wrong population

12.	Nur Azurah Abd Ghani. (2019). A novel approach to manipulate intestinal homeostasis in primary dysmenorrhoea women: A randomised controlled trial (NCT04119011) [Dataset]. https://clinicaltrials.gov/study/NCT04119011	Study not published yet
13.	Özcan, H., Oskay, Ü., & Bodur, A. F. (2019). Effects of kefir on quality of life and sleep disturbances in postmenopausal women. Holistic Nursing Practice, 33(4), 207–213. https://doi.org/10.1097/hnp.0000000000000310	Wrong study design
14.	Rastegar, M., Jahani Shoorab, N., Salari, R., & Saki, A. (2024). The effect of oral probiotic capsules on postpartum depression in primiparous women: A triple-blind randomized control trial. The Iranian Journal of Obstetrics, Gynecology and Infertility, 27(3), 64–75. https://doi.org/10.22038/ijogi.2024.79061.6047	Language restriction (Persian)
15.	Shafie, M., Homayouni Rad, A., Mohammad-Alizadeh-Charandabi, S., & Mirghafourvand, M. (2022). The effect of probiotics on mood and sleep quality in postmenopausal women: A triple-blind randomized controlled trial. Clinical Nutrition ESPEN, 50, 15–23. https://doi.org/10.1016/j.clnesp.2022.06.005	Wrong comparator
16.	Société des Produits Nestlé. (2020). Investigating a probiotic on mothers' mood and stress. *Clinicaltrials.Gov*.	Study not published yet
17.	Stevens, A. J., Heiwari, T. M., Rich, F. J., Bradley, H. A., Gur, T. L., Galley, J. D., Kennedy, M. A., Dixon, L. A., Mulder, R. T., & Rucklidge, J. J. (2024). Randomised control trial indicates micronutrient supplementation may support a more robust maternal microbiome for women with antenatal depression during pregnancy. Clinical Nutrition, 43(11), 120–132. https://doi.org/10.1016/j.clnu.2024.09.004	Wrong intervention
18.	Yurt, M., Mercanlıgil, S. M., & Kabaran, S. (2020). Effect of dairy products intake in women with premenstrual syndrome: A randomized controlled trial. Progress in Nutrition, 22(1), 137–145.	Wrong intervention

Table S3. Results of meta-regressions on duration of treatment (weeks) for depression and anxiety (random-effects)

Outcome	Covariate	Coefficient	SE	95% CI (Lower, Upper)	Z-value	p-value	Tau^2	I^2 (%)	R^2 analog	k (studies)
Depression	Intercept	-1.8822	0.7402	-3.3329, -0.4314	-2.54	0.011	0.7392	91.78	0.00	8
	Duration	0.0785	0.0507	-0.0209, 0.1778	1.55	0.122				
Anxiety	Intercept	-1.7038	0.7379	-3.1501, -0.2576	-2.31	0.021	0.5189	86.21	0.03	5
	Duration	0.0621	0.0573	-0.0502, 0.1743	1.08	0.278				

CI = Confidence interval; SE = Standard error
