# Supplementary material for: Efficacy of Gut Microbiome-Targeted Interventions on Mental Health Symptoms in Women Across Key Hormonal Life Stages: A Systematic Review and Meta-Analysis of Randomized Controlled Trials
Source: Healthcare (Basel). 2025 Nov 10;13(22):2851. doi: 10.3390/healthcare13222851 (PMC12652156; doi:10.3390/healthcare13222851)
Supplement: Supplementary file 1 [file healthcare-13-02851-s001.zip › healthcare-3934528-Supplementary File 2.pdf]

Full electronic search strategy Pubmed/MEDLINE:

Search: **(probiotics OR prebiotics OR synbiotics OR psychobiotics OR postbiotics OR paraprobiotics OR parabiotics OR fermented foods OR yogurt OR yoghurt OR kimchi OR kombucha OR kefir OR sauerkraut OR fermented milk products OR cultured milk products OR microbiome OR microbiota OR gut flora OR intestin* flora OR gut-brain axis OR gut dysbiosis) AND (premenstrual OR menstrua* OR premenstrual disorders OR premenstrual syndrome OR premenstrual symptoms OR PMS OR dysmenorrhea OR luteal phase OR follicular phase OR menarche OR perinatal* OR pregnancy OR postpartum OR PPD OR PND OR prenatal* OR postnatal* OR antenatal* OR menopaus* OR perimenopause*) AND (mental health OR mental well-being OR mental wellbeing OR psychological well-being OR psychological wellbeing OR depression OR anxiety OR emotional wellbeing OR emotional well-being OR psychological distress OR mood* OR stress) AND (randomized controlled trial OR randomised controlled trial OR RCT)**

("probiotic s"[All Fields] OR "probiotical"[All Fields] OR "probiotics"[MeSH Terms] OR "probiotics"[All Fields] OR "probiotic"[All Fields] OR ("prebiotically"[All Fields] OR "prebiotics"[Supplementary Concept] OR "prebiotics"[All Fields] OR "prebiotic"[All Fields] OR "prebiotics"[MeSH Terms]) OR ("synbiotics"[MeSH Terms] OR "synbiotics"[All Fields] OR "synbiotic"[All Fields]) OR ("psychobiotic"[All Fields] OR "psychobiotics"[All Fields]) OR ("postbiotic"[All Fields] OR "postbiotics"[All Fields]) OR "paraprobiotics"[All Fields] OR "parabiotics"[All Fields] OR ("fermented foods"[MeSH Terms] OR ("fermented"[All Fields] AND "foods"[All Fields]) OR "fermented foods"[All Fields]) OR ("yoghurts"[All Fields] OR "yogurt"[MeSH Terms] OR "yogurt"[All Fields] OR "yoghurt"[All Fields] OR "yogurts"[All Fields]) OR ("yoghurts"[All Fields] OR "yogurt"[MeSH Terms] OR "yogurt"[All Fields] OR "yoghurt"[All Fields] OR "yogurts"[All Fields]) OR ("kimchi"[All Fields] OR "kimchis"[All Fields]) OR "kombucha"[All Fields] OR ("kefir"[MeSH Terms] OR "kefir"[All Fields] OR "kefirs"[All Fields]) OR ("sauerkraut"[All Fields] OR "sauerkrauts"[All Fields]) OR ("cultured milk products"[MeSH Terms] OR ("cultured"[All Fields] AND "milk"[All Fields] AND "products"[All Fields]) OR "cultured milk products"[All Fields] OR ("fermented"[All Fields] AND "milk"[All Fields] AND "products"[All Fields]) OR "fermented milk products"[All Fields]) OR ("cultured milk products"[MeSH Terms] OR ("cultured"[All Fields] AND "milk"[All Fields] AND "products"[All Fields]) OR "cultured milk products"[All Fields]) OR ("microbiome s"[All Fields] OR "microbiomic"[All Fields] OR "microbiomics"[All Fields] OR "microbiota"[MeSH Terms] OR "microbiota"[All Fields] OR "microbiome"[All Fields] OR "microbiomes"[All Fields]) OR ("microbiota"[MeSH Terms] OR "microbiota"[All Fields] OR "microbiotas"[All Fields] OR "microbiota s"[All Fields] OR "microbiotae"[All Fields]) OR ("gastrointestinal microbiome"[MeSH Terms] OR ("gastrointestinal"[All Fields] AND "microbiome"[All Fields]) OR "gastrointestinal microbiome"[All Fields] OR ("gut"[All Fields] AND "flora"[All Fields]) OR "gut flora"[All Fields]) OR ("intestin*"[All Fields] AND ("flora"[All Fields] OR "florae"[All Fields] OR "floras"[All Fields])) OR ("brain gut axis"[MeSH Terms] OR ("brain gut"[All Fields] AND "axis"[All Fields]) OR "brain gut axis"[All Fields] OR ("gut"[All Fields] AND "brain"[All Fields] AND "axis"[All Fields]) OR "gut brain axis"[All Fields]) OR (("gut"[Journal] OR "gut"[All Fields]) AND ("dysbiosis"[MeSH Terms] OR "dysbiosis"[All Fields] OR "dysbioses"[All Fields]))) AND ("premenstrual"[All Fields] OR "premenstrually"[All Fields] OR "menstrua*"[All Fields] OR (("premenstrual"[All Fields] OR "premenstrually"[All Fields]) AND ("disease"[MeSH Terms] OR "disease"[All Fields] OR "disorder"[All Fields] OR "disorders"[All Fields] OR "disorder s"[All Fields] OR "disordes"[All Fields])) OR ("premenstrual syndrome"[MeSH Terms] OR ("premenstrual"[All Fields] AND "syndrome"[All Fields]) OR "premenstrual syndrome"[All Fields]) OR (("premenstrual"[All Fields] OR "premenstrually"[All Fields]) AND ("diagnosis"[MeSH Subheading] OR "diagnosis"[All Fields] OR

"symptoms"[All Fields] OR "diagnosis"[MeSH Terms] OR "symptom"[All Fields] OR "symptom s"[All Fields] OR "symptomes"[All Fields])) OR "PMS"[All Fields] OR ("dysmenorrhea"[MeSH Terms] OR "dysmenorrhea"[All Fields] OR "dysmenorrheas"[All Fields] OR "dysmenorrhoea"[All Fields]) OR ("luteal phase"[MeSH Terms] OR ("luteal"[All Fields] AND "phase"[All Fields]) OR "luteal phase"[All Fields]) OR ("follicular phase"[MeSH Terms] OR ("follicular"[All Fields] AND "phase"[All Fields]) OR "follicular phase"[All Fields]) OR ("menarch"[All Fields] OR "menarchal"[All Fields] OR "menarche"[MeSH Terms] OR "menarche"[All Fields] OR "menarcheal"[All Fields] OR "menarches"[All Fields]) OR "perinatal*"[All Fields] OR ("pregnancy"[MeSH Terms] OR "pregnancy"[All Fields] OR "pregnancies"[All Fields] OR "pregnancy s"[All Fields]) OR ("postpartum period"[MeSH Terms] OR ("postpartum"[All Fields] AND "period"[All Fields]) OR "postpartum period"[All Fields] OR "postpartum"[All Fields]) OR "PPD"[All Fields] OR "PND"[All Fields] OR "prenatal*"[All Fields] OR "postnatal*"[All Fields] OR "antenatal*"[All Fields] OR "menopaus*"[All Fields] OR "perimenopause*"[All Fields]) AND ("mental health"[MeSH Terms] OR ("mental"[All Fields] AND "health"[All Fields]) OR "mental health"[All Fields] OR ("mental health"[MeSH Terms] OR ("mental"[All Fields] AND "health"[All Fields]) OR "mental health"[All Fields] OR ("mental"[All Fields] AND "well"[All Fields]) OR "mental well being"[All Fields]) OR (("mental"[All Fields] OR "mentalities"[All Fields] OR "mentality"[All Fields] OR "mentalization"[MeSH Terms] OR "mentalization"[All Fields] OR "mentalizing"[All Fields] OR "mentalize"[All Fields] OR "mentalized"[All Fields] OR "mentally"[All Fields]) AND "wellbeing"[All Fields]) OR ("psychological well being"[MeSH Terms] OR ("psychological"[All Fields] AND "well being"[All Fields]) OR "psychological well being"[All Fields] OR ("psychological"[All Fields] AND "well"[All Fields]) OR "psychological well being"[All Fields]) OR (("psychologic"[All Fields] OR "psychological"[All Fields] OR "psychologically"[All Fields] OR "psychologization"[All Fields] OR "psychologized"[All Fields] OR "psychologizing"[All Fields]) AND "wellbeing"[All Fields]) OR ("depressed"[All Fields] OR "depression"[MeSH Terms] OR "depression"[All Fields] OR "depressions"[All Fields] OR "depression s"[All Fields] OR "depressive disorder"[MeSH Terms] OR ("depressive"[All Fields] AND "disorder"[All Fields]) OR "depressive disorder"[All Fields] OR "depressivity"[All Fields] OR "depressive"[All Fields] OR "depressively"[All Fields] OR "depressiveness"[All Fields] OR "depressives"[All Fields]) OR ("anxiety"[MeSH Terms] OR "anxiety"[All Fields] OR "anxieties"[All Fields] OR "anxiety s"[All Fields]) OR (("emoting"[All Fields] OR "emotion s"[All Fields] OR "emotions"[MeSH Terms] OR "emotions"[All Fields] OR "emotion"[All Fields] OR "emotional"[All Fields] OR "emotive"[All Fields]) AND "wellbeing"[All Fields]) OR (("emoting"[All Fields] OR "emotion s"[All Fields] OR "emotions"[MeSH Terms] OR "emotions"[All Fields] OR "emotion"[All Fields] OR "emotional"[All Fields] OR "emotive"[All Fields]) AND ("health"[MeSH Terms] OR "health"[All Fields] OR "well"[All Fields] OR "well being"[All Fields])) OR ("psychological distress"[MeSH Terms] OR ("psychological"[All Fields] AND "distress"[All Fields]) OR "psychological distress"[All Fields]) OR "mood*"[All Fields] OR ("stress"[All Fields] OR "stressed"[All Fields] OR "stresses"[All Fields] OR "stressful"[All Fields] OR "stressfulness"[All Fields] OR "stressing"[All Fields])) AND ("randomized controlled trial"[Publication Type] OR "randomized controlled trials as topic"[MeSH Terms] OR "randomized controlled trial"[All Fields] OR "randomised controlled trial"[All Fields] OR ("randomized controlled trial"[Publication Type] OR "randomized controlled trials as topic"[MeSH Terms] OR "randomised controlled trial"[All Fields] OR "randomized controlled trial"[All Fields]) OR "RCT"[All Fields])

Translations

probiotics: "probiotic's"[All Fields] OR "probiotical"[All Fields] OR "probiotics"[MeSH Terms] OR "probiotics"[All Fields] OR "probiotic"[All Fields]

prebiotics: "prebiotically"[All Fields] OR "prebiotics"[Supplementary Concept] OR "prebiotics"[All Fields] OR "prebiotic"[All Fields] OR "prebiotics"[MeSH Terms]

synbiotics: "synbiotics"[MeSH Terms] OR "synbiotics"[All Fields] OR "synbiotic"[All Fields]

psychobiotics: "psychobiotic"[All Fields] OR "psychobiotics"[All Fields]

postbiotics: "postbiotic"[All Fields] OR "postbiotics"[All Fields]

fermented foods: "fermented foods"[MeSH Terms] OR ("fermented"[All Fields] AND "foods"[All Fields]) OR "fermented foods"[All Fields]

yogurt: "yoghurts"[All Fields] OR "yogurt"[MeSH Terms] OR "yogurt"[All Fields] OR "yoghurt"[All Fields] OR "yogurts"[All Fields]

yoghurt: "yoghurts"[All Fields] OR "yogurt"[MeSH Terms] OR "yogurt"[All Fields] OR "yoghurt"[All Fields] OR "yogurts"[All Fields]

kimchi: "kimchi"[All Fields] OR "kimchis"[All Fields]

kefir: "kefir"[MeSH Terms] OR "kefir"[All Fields] OR "kefirs"[All Fields]

sauerkraut: "sauerkraut"[All Fields] OR "sauerkrauts"[All Fields]

fermented milk products: "cultured milk products"[MeSH Terms] OR ("cultured"[All Fields] AND "milk"[All Fields] AND "products"[All Fields]) OR "cultured milk products"[All Fields] OR ("fermented"[All Fields] AND "milk"[All Fields] AND "products"[All Fields]) OR "fermented milk products"[All Fields]

cultured milk products: "cultured milk products"[MeSH Terms] OR ("cultured"[All Fields] AND "milk"[All Fields] AND "products"[All Fields]) OR "cultured milk products"[All Fields]

microbiome: "microbiome's"[All Fields] OR "microbiomic"[All Fields] OR "microbiomics"[All Fields] OR "microbiota"[MeSH Terms] OR "microbiota"[All Fields] OR "microbiome"[All Fields] OR "microbiomes"[All Fields]

microbiota: "microbiota"[MeSH Terms] OR "microbiota"[All Fields] OR "microbiotas"[All Fields] OR "microbiota's"[All Fields] OR "microbiotae"[All Fields]

gut flora: "gastrointestinal microbiome"[MeSH Terms] OR ("gastrointestinal"[All Fields] AND "microbiome"[All Fields]) OR "gastrointestinal microbiome"[All Fields] OR ("gut"[All Fields] AND "flora"[All Fields]) OR "gut flora"[All Fields]

flora: "flora"[All Fields] OR "florae"[All Fields] OR "floras"[All Fields]

gut-brain axis: "brain-gut axis"[MeSH Terms] OR ("brain-gut"[All Fields] AND "axis"[All Fields]) OR "brain-gut axis"[All Fields] OR ("gut"[All Fields] AND "brain"[All Fields] AND "axis"[All Fields]) OR "gut brain axis"[All Fields]

gut: "Gut"[Journal:__jid2985108R] OR "gut"[All Fields]

dysbiosis: "dysbiosis"[MeSH Terms] OR "dysbiosis"[All Fields] OR "dysbioses"[All Fields]

premenstrual: "premenstrual"[All Fields] OR "premenstrually"[All Fields]

premenstrual: "premenstrual"[All Fields] OR "premenstrually"[All Fields]

disorders: "disease"[MeSH Terms] OR "disease"[All Fields] OR "disorder"[All Fields] OR "disorders"[All Fields] OR "disorder's"[All Fields] OR "disordes"[All Fields]

premenstrual syndrome: "premenstrual syndrome"[MeSH Terms] OR ("premenstrual"[All Fields] AND "syndrome"[All Fields]) OR "premenstrual syndrome"[All Fields]

premenstrual: "premenstrual"[All Fields] OR "premenstrually"[All Fields]

symptoms: "diagnosis"[Subheading] OR "diagnosis"[All Fields] OR "symptoms"[All Fields] OR "diagnosis"[MeSH Terms] OR "symptom"[All Fields] OR "symptom's"[All Fields] OR "symptomes"[All Fields]

dysmenorrhea: "dysmenorrhea"[MeSH Terms] OR "dysmenorrhea"[All Fields] OR "dysmenorrheas"[All Fields] OR "dysmenorrhoea"[All Fields]

luteal phase: "luteal phase"[MeSH Terms] OR ("luteal"[All Fields] AND "phase"[All Fields]) OR "luteal phase"[All Fields]

follicular phase: "follicular phase"[MeSH Terms] OR ("follicular"[All Fields] AND "phase"[All Fields]) OR "follicular phase"[All Fields]

menarche: "menarch"[All Fields] OR "menarchal"[All Fields] OR "menarche"[MeSH Terms] OR "menarche"[All Fields] OR "menarcheal"[All Fields] OR "menarches"[All Fields]

pregnancy: "pregnancy"[MeSH Terms] OR "pregnancy"[All Fields] OR "pregnancies"[All Fields] OR "pregnancy's"[All Fields]

postpartum: "postpartum period"[MeSH Terms] OR ("postpartum"[All Fields] AND "period"[All Fields]) OR "postpartum period"[All Fields] OR "postpartum"[All Fields]

mental health: "mental health"[MeSH Terms] OR ("mental"[All Fields] AND "health"[All Fields]) OR "mental health"[All Fields]

mental well-being: "mental health"[MeSH Terms] OR ("mental"[All Fields] AND "health"[All Fields]) OR "mental health"[All Fields] OR ("mental"[All Fields] AND "well"[All Fields]) OR "mental well being"[All Fields]

mental: "mental"[All Fields] OR "mentalities"[All Fields] OR "mentality"[All Fields] OR "mentalization"[MeSH Terms] OR "mentalization"[All Fields] OR "mentalizing"[All Fields] OR "mentalize"[All Fields] OR "mentalized"[All Fields] OR "mentally"[All Fields]

psychological well-being: "psychological well-being"[MeSH Terms] OR ("psychological"[All Fields] AND "well-being"[All Fields]) OR "psychological well-being"[All Fields] OR ("psychological"[All Fields] AND "well"[All Fields]) OR "psychological well being"[All Fields]

psychological: "psychologic"[All Fields] OR "psychological"[All Fields] OR "psychologically"[All Fields] OR "psychologization"[All Fields] OR "psychologized"[All Fields] OR "psychologizing"[All Fields]

depression: "depressed"[All Fields] OR "depression"[MeSH Terms] OR "depression"[All Fields] OR "depressions"[All Fields] OR "depression's"[All Fields] OR "depressive disorder"[MeSH Terms] OR ("depressive"[All Fields] AND "disorder"[All Fields]) OR "depressive disorder"[All Fields] OR

"depressivity"[All Fields] OR "depressive"[All Fields] OR "depressively"[All Fields] OR "depressiveness"[All Fields] OR "depressives"[All Fields]

anxiety: "anxiety"[MeSH Terms] OR "anxiety"[All Fields] OR "anxieties"[All Fields] OR "anxiety's"[All Fields]

emotional: "emoting"[All Fields] OR "emotion's"[All Fields] OR "emotions"[MeSH Terms] OR "emotions"[All Fields] OR "emotion"[All Fields] OR "emotional"[All Fields] OR "emotive"[All Fields]

emotional: "emoting"[All Fields] OR "emotion's"[All Fields] OR "emotions"[MeSH Terms] OR "emotions"[All Fields] OR "emotion"[All Fields] OR "emotional"[All Fields] OR "emotive"[All Fields]

well-being: "health"[MeSH Terms] OR "health"[All Fields] OR ("well"[All Fields]) OR "well being"[All Fields]

psychological distress: "psychological distress"[MeSH Terms] OR ("psychological"[All Fields] AND "distress"[All Fields]) OR "psychological distress"[All Fields]

stress: "stress"[All Fields] OR "stressed"[All Fields] OR "stresses"[All Fields] OR "stressful"[All Fields] OR "stressfulness"[All Fields] OR "stressing"[All Fields]

randomized controlled trial: "randomized controlled trial"[Publication Type] OR "randomized controlled trials as topic"[MeSH Terms] OR "randomized controlled trial"[All Fields] OR "randomised controlled trial"[All Fields]

randomised controlled trial: "randomized controlled trial"[Publication Type] OR "randomized controlled trials as topic"[MeSH Terms] OR "randomised controlled trial"[All Fields] OR "randomized controlled trial"[All Fields]
